# Unilateral Lateral Crural Overlay Technique for the Correction of Nasal Tip Asymmetry: A Case Report

**DOI:** 10.7759/cureus.112383

**Published:** 2026-07-10

**Authors:** Alexandros Georgolios, Vasiliki Zisi, Michalis Tsounis, Georgios V Psychogios

**Affiliations:** 1 Otorhinolaryngology, Head and Neck Surgery, University Hospital of Ioannina, Ioannina, GRC; 2 School of Medicine, University of Ioannina, Ioannina, GRC; 3 Otorhinolaryngology (ENT Surgery), Private Practice, Athens, GRC

**Keywords:** lateral crura overlay, lower lateral cartilages, nasal tip asymmetry, nasal valve, rhinoplasty, septal deviation

## Abstract

The aim of the present study is the presentation of a case of nasal tip asymmetry and the evaluation of the unilateral application of the lateral crural overlay technique as a targeted surgical approach. A clinical case of a patient presenting with both functional and esthetic nasal disturbance is described. Following confirmation of unilateral anatomical imbalance of the lower lateral cartilages, open rhinoplasty was performed in combination with unilateral application of the technique. Postoperative evaluation demonstrated improvement in the deviation of the nasal tip from the midline, as well as preservation of nasal valve function. The results of postoperative assessment indicate that unilateral application of the aforementioned technique may constitute an effective and individualized approach in selected cases of nasal tip asymmetry.

## Introduction

The nose occupies a central position on the face and, in combination with its complex anatomy and functional role, surgical correction of nasal tip asymmetry constitutes one of the most challenging problems in facial plastic surgery [1]. Primary anatomical imbalances of the lower lateral cartilages, as well as septal deviation, represent key factors that contribute to the final configuration of the nasal tip [2,3].

A fundamental component of successful reconstruction is the appropriate selection of surgical technique, which should be individualized and based on the underlying anatomical abnormality [4-6]. The lateral crural overlay technique constitutes one of the main options in cases associated with the length, curvature, and dominance of the lower lateral cartilages [4,7,8], and is more commonly applied using a bilateral approach, with the primary goal of achieving overall reshaping of the nasal tip [2]. Notably, unilateral application of this technique may represent a more focused and individualized alternative in cases of isolated unilateral anatomical imbalance. Compared with the conventional bilateral approach, unilateral lateral crural overlay allows selective correction of the affected side while preserving the contralateral lower lateral cartilage, potentially reducing unnecessary surgical manipulation and maintaining structural integrity. This targeted strategy may offer particular clinical value in carefully selected patients with nasal tip asymmetry [2,4].

However, in cases where asymmetry is attributed to unilateral anatomical imbalance, unilateral application of the aforementioned technique may provide a more targeted and conservative correction, limiting the extent of surgical intervention. The aim of the present study is the presentation of a case of nasal tip asymmetry, as well as the demonstration of the unilateral application of the lateral crural overlay technique as an individualized and effective surgical approach in cases of unilateral anatomical imbalance.

## Case presentation

A 45-year-old male patient presented with a primary complaint of impaired nasal breathing, accompanied by esthetic concern due to asymmetry of the nasal tip. Clinical examination was performed, which confirmed deviation of the nasal tip from the midline, with evident asymmetry of the alar structures attributed to dominance of the right lower lateral cartilage, as well as septal deviation.

**Figure 1:**
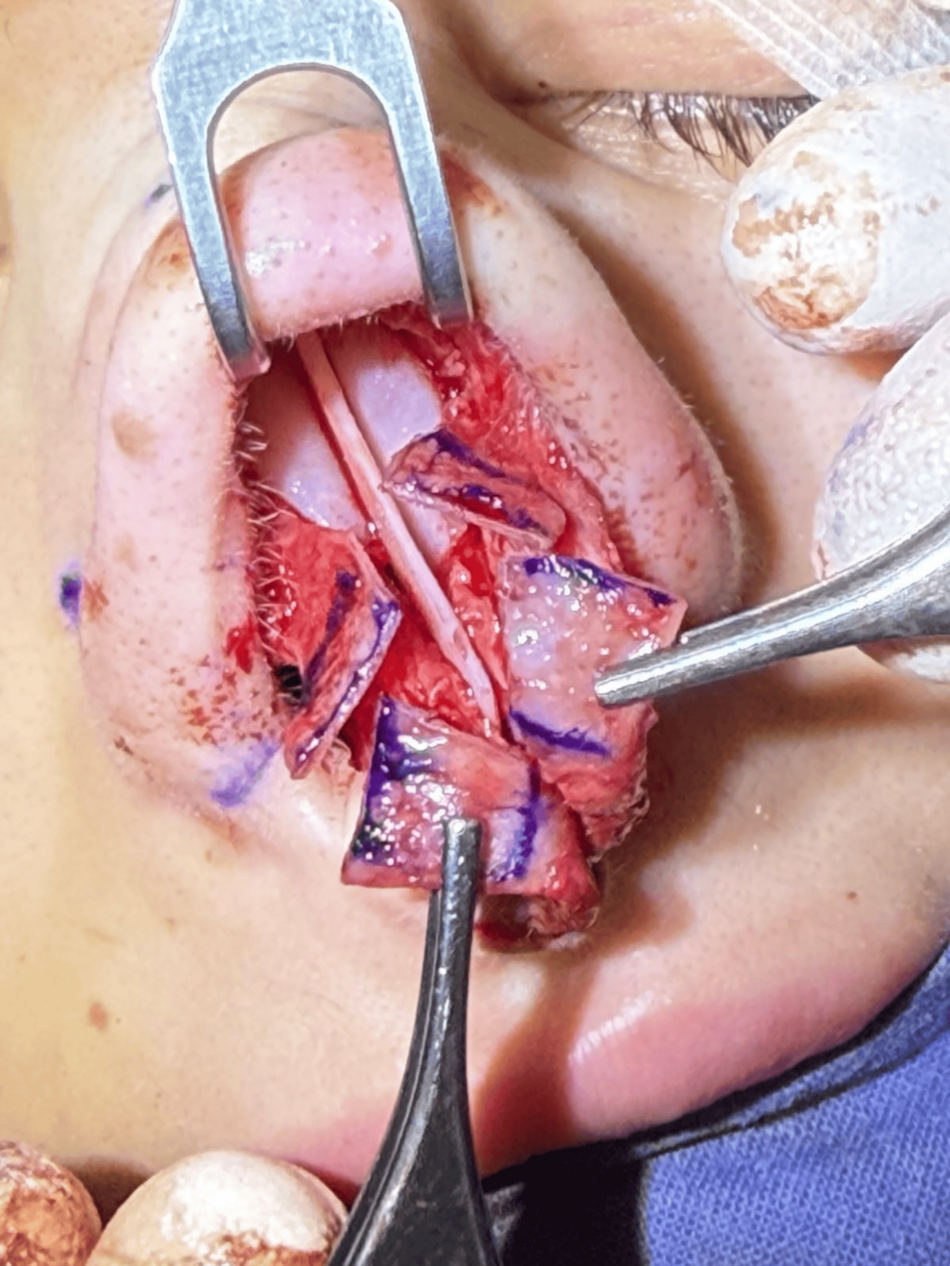
Intraoperative view illustrating unilateral application of the lateral crural overlay technique with controlled shortening and overlap of the dominant lower lateral cartilage.

**Figure 2:**
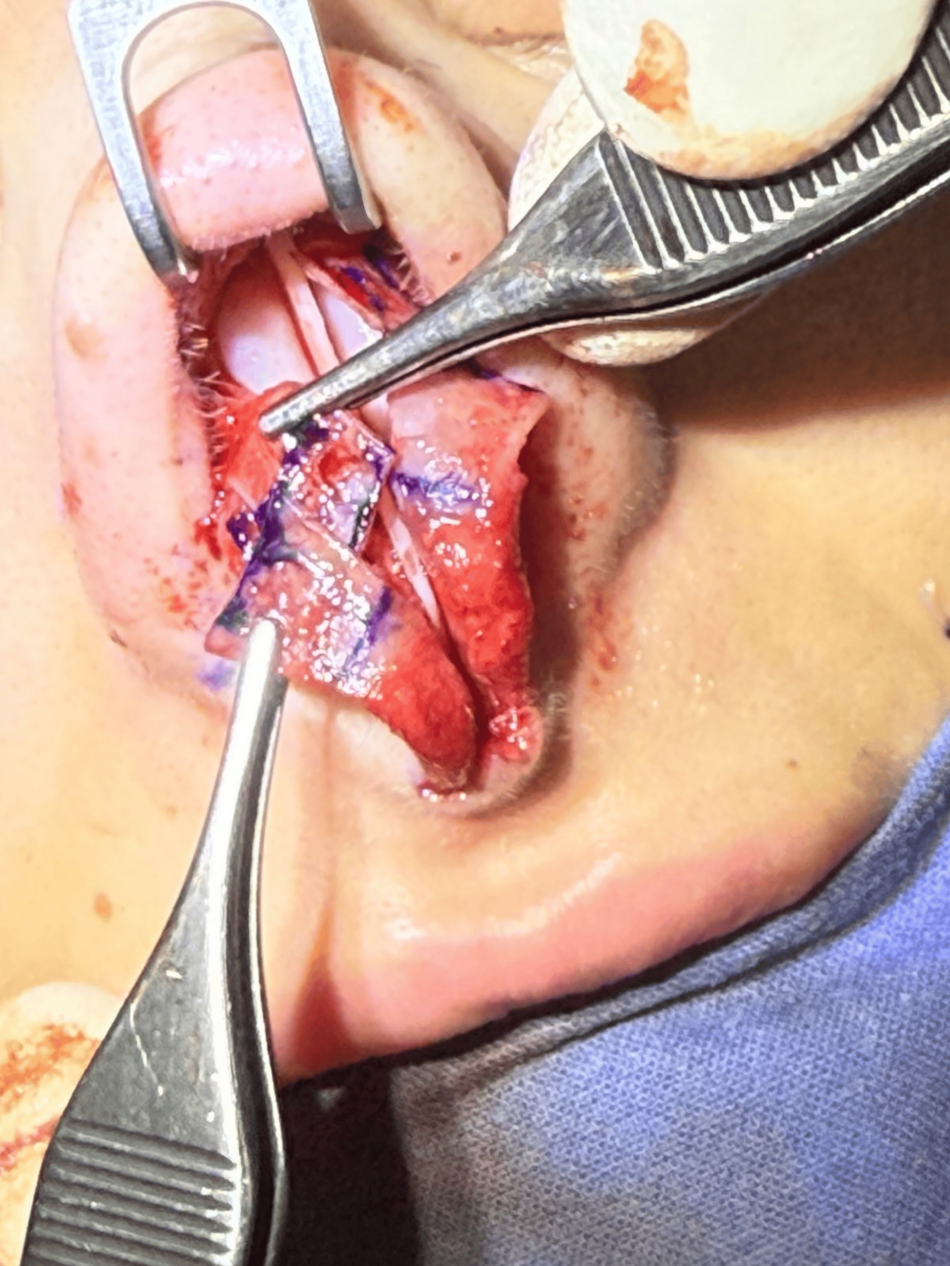
Additional intraoperative view illustrating unilateral application of the lateral crural overlay technique with controlled shortening and overlap of the dominant lower lateral cartilage.

**Figure 3:**
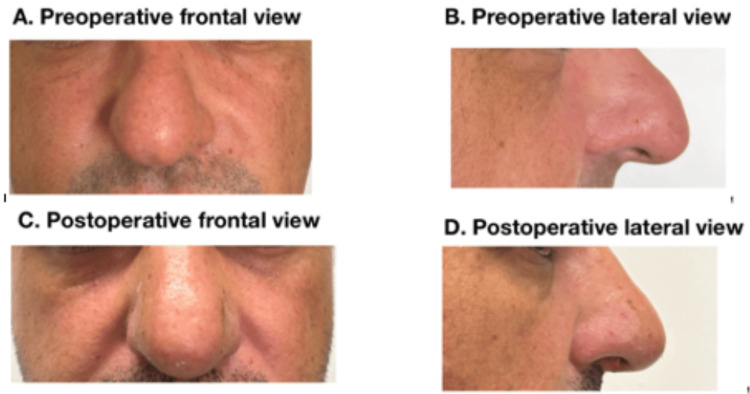
Standardized preoperative and postoperative external photographs. Preoperative frontal (A) and lateral (B) views demonstrating nasal tip asymmetry with deviation from the midline. Postoperative frontal (C) and lateral (D) views showing correction of nasal tip asymmetry and improved nasal tip alignment and symmetry.

Following endoscopic evaluation, functional impairment of nasal breathing was confirmed, and preoperative photographic documentation of the findings was obtained. Preoperative assessment using the Nasal Obstruction Symptom Evaluation (NOSE) questionnaire [9] demonstrated severe nasal obstruction, with a raw score of 18/20 (standardized score 90/100). At postoperative follow-up, the NOSE score improved to 0/20 (standardized score 0/100), indicating complete resolution of nasal obstruction symptoms. The patient underwent open rhinoplasty under general anesthesia. Initially, correction of the deviated nasal septum was performed; however, during the surgical procedure, persistent asymmetry was observed due to unilateral dominance of the right lower lateral cartilage. To restore the anatomical imbalance and achieve symmetry between the two sides, unilateral application of the lateral crural overlay technique was performed (Figures 1, 2). Preoperative and postoperative standardized external photographs are presented in Figure 3.

Specifically, the longer cartilage was subjected to careful shortening, followed by controlled overlap and suturing of its two distinct segments [4]. The dominant right lower lateral cartilage was shortened and stabilized with polydioxanone (PDS) sutures following controlled overlap of the cartilage segments. During septal correction, an L-strut of 15 mm was preserved, with 15 mm maintained in the keystone area to ensure dorsal and caudal septal support. This intervention aimed at selective shortening of the affected cartilage and reduction of its projection, with the ultimate goal of restoring nasal tip symmetry while preserving the integrity of the nasal valve.

Postoperative follow-up lasted 10 months. During re-evaluation, a clear improvement and realignment of the nasal tip relative to the midline was observed, along with balanced projection. In addition, from a functional perspective, satisfactory restoration of nasal breathing was noted, with no evidence of complications.

## Discussion

The nasal tip represents a complex anatomical and functional unit of the nose, as its morphology and stability depend closely on the interaction between cartilaginous structures and soft tissues [5,10]. The lower lateral cartilages play a multifactorial role in the configuration of the nasal tip. Specifically, they influence its projection and symmetry, while at the same time they are essential for maintaining the function of the external nasal valve [5,11].

Even minor variations in the position, length, or curvature of the lower lateral cartilages may lead to significant and clinically evident differences in the nasal tip, making surgical management particularly demanding and highly individualized [1]. The factors associated with nasal tip asymmetry are multiple, including intrinsic asymmetry of the lower lateral cartilages, morphological variations, as well as the presence of associated deformities of the nasal septum [3].

However, cartilaginous abnormalities should be recognized and managed as an independent cause of asymmetry, since correction of septal deviation alone does not always lead to restoration of nasal tip symmetry [3]. Therefore, successful correction requires the selection of an appropriate surgical technique, tailored to the underlying anatomical abnormality [6].

Among the available techniques, the lateral crural overlay technique is widely used in cases where one lower lateral cartilage is dominant, as it allows reshaping and shortening of the cartilage, leading to the desired esthetic outcome [4]. In most cases, the technique is applied bilaterally, particularly when the primary goal is global correction and reshaping of the nasal tip [2].

Alternative techniques have also been described for the management of nasal tip asymmetry. Cephalic trim is commonly used in patients with bulky nasal tips and may improve tip definition. However, excessive resection can weaken the lower lateral cartilages and potentially compromise structural support. Lateral crural steal may improve tip projection and rotation through redistribution of the existing cartilage, whereas septal extension grafts provide strong structural support and precise tip control. In contrast, unilateral lateral crural overlay offers a more targeted approach in cases of isolated unilateral lower lateral cartilage dominance, correcting asymmetry while preserving the contralateral side and maintaining nasal valve function [5,6,11].

However, when asymmetry is unilateral and localized, a bilateral approach may lead to overcorrection or unnecessary modification of otherwise normal anatomical structures. In such cases, unilateral application of the technique represents a more targeted surgical approach, as it allows correction of the affected cartilage while preserving the integrity of the healthy contralateral side.

This approach not only ensures a satisfactory esthetic outcome, but also maintains the balance of the nasal tip and minimizes the risk of functional complications, such as collapse of the external nasal valve [11,12].

In the present case, the persistent asymmetry following septoplasty was attributed to unilateral dominance of one lower lateral cartilage, which rendered unilateral application of the technique the most appropriate option. The technique was applied as a complementary surgical intervention, with the aim of selectively correcting the underlying cartilaginous asymmetry. Its application resulted in successful correction of the asymmetry, while simultaneously preserving nasal valve function.

Although the outcome is encouraging, the present study is limited by the fact that it represents a single clinical case. Further studies with a larger number of patients are required in order to confirm the effectiveness and broader applicability of this surgical approach.

## Conclusions

Successful correction of nasal tip asymmetry requires precise identification and targeted management of the underlying anatomical cause. Unilateral application of the lateral crural overlay technique may represent a particularly useful complementary surgical approach in selected cases of nasal tip asymmetry resulting from unilateral cartilaginous imbalance.

In the present case, the technique allowed selective correction of the asymmetry, while simultaneously preserving the structural and functional integrity of the nose. Effective management of such cases requires accurate anatomical assessment and individualized surgical planning.
